# Prevalence of Metastasis and Involvement of Level IV and V in Oral Squamous Cell Carcinoma: A Systematic Review

**DOI:** 10.7759/cureus.20255

**Published:** 2021-12-07

**Authors:** Ahmad A Altuwaijri, Turki M Aldrees, Mohammed A Alessa

**Affiliations:** 1 Department of Otolaryngology-Head and Neck Surgery, College of Medicine, King Saud University, Riyadh, SAU; 2 Department of Otolaryngology-Head and Neck Surgery, Security Forces Hospital, Riyadh, SAU; 3 Department of Otolaryngology-Head and Neck Surgery, College of Medicine, Prince Sattam Bin Abdulaziz University, Alkharj, SAU

**Keywords:** supraomohyoid neck dissection, selective neck dissection, skip metastasis, level v, level iv, metastasis, elective neck dissection, oral squamous cell carcinoma

## Abstract

The occurrence of occult metastases in oral cavity squamous cell carcinoma (OSCC) to lower levels in the neck (levels IV and V) or development of skip metastases that bypass the upper neck levels (levels I to III) and go directly to level IV or V is common. This challenges the efficacy of conventional neck dissection approaches in the treatment of OSCC. Therefore, the decision to include lower levels cervical nodes during elective neck dissection of OSCC remains controversial.

This systematic review was designed to assess the prevalence of level IV and/or V involvement or skip metastases in patients with the clinically negative neck (cN0) or positive (cN+) oral squamous cell carcinoma (OSCC). We searched for studies published between December 2000 and December 2020. Potentially relevant abstracts and full-text articles were screened, and data from the studies were extracted. Quality was rated using the Newcastle Ottawa Scale (NOS) criteria.

In total, 802 abstracts and 227 full-text articles were screened, and 32 studies were included in this analysis. The prevalence of metastasis ranged from 1.8% to 66.0%. The incidence for skip metastasis to level IV or V was low, reaching 8.5%. Evidence favored elective neck dissection, including levels I to III, in selected patients with OSCC and patients with cN0 or cN+ neck. The literature was non-conclusive on the recommendation for inclusion of lower levels.

## Introduction and background

Oral squamous cell carcinoma (OSCC), constituted by a broad range of tumors with diverse etiologies, is a life-threatening malignant tumor that ranks as the sixth most common cancer by incidence, with 500,000 new cases reported worldwide annually, accounting for 32%-40% of all head and neck cancers [1,2]. It can metastasize to cervical lymph nodes via lymphatic vessels [2,3], with neck metastasis being the most important prognostic factor which affected survival by a nearly 50% decline [4]. The incidence of clinical cervical metastases from OSCC has been found to occur in as many as 40% of cases [5]. Moreover, occult regional lymph node metastases incidence detected using histopathological and immunohistochemical methods was found to range between 15% and 34% [6] among patients without clinical or radiologic evidence of lymph node metastases preoperatively.

Selective neck dissection (SND), which removes lymph node groups at designated anatomic levels (I-III), is accepted as the standard of care for the management of regional disease in OSCC patients with clinically positive node (cN+) involvement [7,8], as well as the standard elective procedure for clinically node-negative (cN0) patients or those with microscopic disease [9,10], resulting in improved quality of life and a lower likelihood of orofacial complication or shoulder dysfunction compared to other modalities, including comprehensive neck dissection, such as modified radical neck dissection (MRND) or radical neck dissection (RND) [11,12]. However, several studies have concluded that supraomohyoid neck dissection (SOHND, level I-III) is inadequate in patients with OSCC, owing to occult metastasis to neck level IV and that this level should be routinely dissected [13,14].

In view of the controversies surrounding the inclusion of lower levels for dissection, the present study was designed with the objectives of conducting a systematic review of all relevant published literature: (i) to study the prevalence and distribution of metastasis levels and related adverse outcomes in clinically N0 and N+ OSCC; and (ii) to determine the frequency of involvement of levels IV and V, as well as skip metastasis to level IV in patients diagnosed with OCSCC without preoperative evidence of neck involvement. We aimed to summarize the recommendations for routine dissection of lower levels of nodes in patients with OSCC.

## Review

Methodology

Search Strategy

A comprehensive search for all relevant articles published in English between January 2000 and December 2020 was performed using the electronic databases PubMed, Embase, Ovid, Google Scholar, and Science Direct. We included retrospective, prospective, clinical trials, and cross-sectional studies. The key search terms used either alone or in combination were neck dissection, radical neck dissection, cN0 neck, cN+ neck, oral squamous cell carcinoma, skip metastasis, occult metastasis, lymph node management, neck metastasis, oral cavity cancer, and tongue cancer. The references of articles and citations were also searched for additional potentially relevant publications.

Study Eligibility Criteria

All studies that included patients who underwent a neck dissection (ND) of at least levels I through III or I-IV and presented information on clinically node-negative (cN0) and/or clinically node-positive (cN+) necks were eligible for inclusion. The inclusion criteria were as follows: (1) any prospective or retrospective cohort, (2) a study population with the histopathologic diagnosis of OSCC, and (3) full text available in the English language. In addition, studies that reported skip metastasis (metastasis solely at neck level IV or V) were also eligible for inclusion. Exclusion criteria were as follows: (1) studies on patients who underwent treatment other than surgery as primary treatment, such as preoperative radiotherapy and chemotherapy, and (2) studies on recurrent tumors or tumors other than SCC.

Data Extraction

Information regarding patient characteristics, primary tumor site, treatment, sample size, metastasis, authors, publication year, and the country was retrieved from the selected articles. Data were initially extracted and evaluated by two authors (AA, TA). The distributions of the T category, the extent of ND, the subsite of the primary tumor, and nodal metastasis were recorded. A skip metastasis was defined as a positive level IV (or lower) node on final pathology without the involvement of higher levels (i.e., levels I-III). A level IV nodal metastasis coexisting with nodes at other neck levels was assessed separately. We followed the Preferred Reporting Items for Systematic Reviews and Meta-Analyses (PRISMA) guidelines for reporting the included observational studies [15].

Quality Evaluation

The quality of literature was evaluated according to the Newcastle Ottawa Scale (NOS) evaluation criteria [16]. By quality evaluation, 21 references were ranked high, seven references were medium, and only four were ranked low (Table 1).

**Table 1:** The quality rating of included studies using the Newcastle Ottawa Scale (NOS)

Author	Year	NOS quality rating
Silverman [17]	2003	8
Anderson [18]	2002	7
Jena [19]	2013	7
Liao [20]	2011	6
Jayasuriya [21]	2020	8
Haranadha [22]	2018	7
Chheda [23]	2014	7
Kakei [24]	2020	8
Marchiano [25]	2016	4
Givi [26]	2012	5
Pandey [27]	2018	7
Agarwal [28]	2018	3
Mishra [29]	2010	6
Shimura [30]	2019	7
Parikh [31]	2013	6
Jerjes [32]	2010	6
Cariati [33]	2018	7
Patel [34]	2019	5
Lodder [35]	2008	5
Lim [36]	2006	6
Kowalski [37]	2002	7
Feng [38]	2013	8
Sivanandan [39]	2004	7
Crean [40]	2003	4
Khafif [41]	2001	6
Balasubramanian [42]	2012	7
Köhler [43]	2018	8
Deo [44]	2007	7
de Vicente [45]	2015	7
Rani [46]	2015	3
Chatterjee [47]	2019	6
Vishak [48]	2014	7

Results

**Figure 1:**
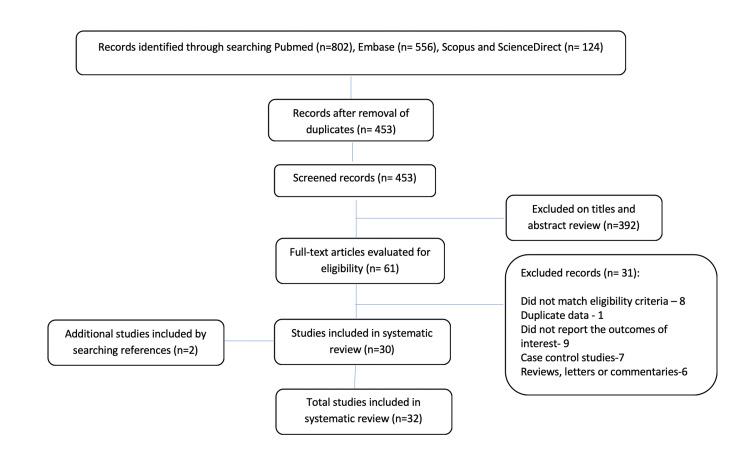
PRISMA flowchart: selection of studies for systematic review PRISMA, Preferred Reporting Items for Systematic Reviews and Meta-Analyses

The search and selection process of the articles is presented in Figure 1. A total of 1482 articles were identified via the database search based on the selection criteria, and two additional articles were later found through reviewing articles and reference lists of retrieved articles. After removing duplicates, 453 articles were screened by their titles and abstracts, and 61 were retained. After full-text revision, 31 articles were excluded (Figure 1). Thus, 32 studies [17-48], all published in English, were included for further analysis.

Description of the Studies

Data of 12,309 patients included in the 32 studies were analyzed. In all studies, cases of level IV or V metastasis and cervical IIb metastasis were confirmed by pathologic examination or other technologies. All studies did not, however, have consistent inclusion criteria and exclusion criteria. Five studies [19,23,28,30,40] reported data from only OSCC patients with cN0, while three [18,21,24] had only data on cN+; five studies [17,29,31,33,35] had mixed data of clinical N0 and N+ cases. The details of the studies included are summarized in Table 2.

**Table 2:** Study characteristics and pattern of lymph node metastasis in oral cavity squamous cell carcinoma SCC, squamous cell carcinoma; HNSCC, head and neck SCC; OSCC, oral cavity SCC; TNM, tumor-node-metastasis staging system; SND, selective neck dissection; SOHND, supraomohyoid neck dissection; SSND, super-selective neck dissection; ESOND, extended supraomohyoid neck dissection; MRND, modified radical neck dissection; RND, radical neck dissection; cN/pN, clinical lymph node status/pathological lymph node status; FOM, floor of mouth; RMT, retromolar trigone; DSS, disease-specific survival; LN, lymph nodes; Ca, cancer; mets, metastasis; Pts, patients.

Author	Year	Region	n	Male %	Primary site	Clinical staging	Metastasis prevalence %	Metastasis level	Treatment given	Recurrence/Survival	Other risk factors	Outcome
Silverman [17]	2003	US	74	55%	HNSCC	TNM	4.40%	N0- 1.6% (in level IIB)	SND Level II	Recurrence- 5.6%	NA	Level V not recommended
					Oral cavity- 47.3%			N1- 11.1% (in level IIB)				
Anderson [18]	2002	US	106	71.70%	Oral cavity- 39.6%	TNM	all N+ve	N1- 54.7%	SOHND I-III	5 year-DSS- 68.8%	NA	NA
								N2a- 4.7%	SND II-IV	Local Recurrence- 12.3%		
								N2b- 26.4%	SND I-IV	Regional recurrence- 4.3%		
								N2c- 13.2%				
								N3- 0.9%				
Jena [19]	2013	India	218	15.60%	Oral ca.	cN0-31.1%	10.4% (occult metastasis)	I- 50 Pts	SOHND	NA	Alcohol	Inconclusive, decision to be based on pre-operative high-risk factors like the site, differentiation, socio-economic status, presence of occult metastasis.
					Buccal mucosa- 53.2%			II- 32 Pts	MRND		Betelnut	
					Gingivobuccal sulcus- 33%		LN metastasis 30.27%	III-15 Pts			Smoking	
								IV- 2 Pts			Tobacco	
								V- 2 Pts				
								Skip metastasis- 1.8%				
Liao [20]	2011	Taiwan	255	94.10%	OSCC	T1-T4	33% (Distant)	IV/V-8.2%	Radical or modified neck dissection I-IV	Local recurrence- 16%	Alcohol	Level IV/V involvement has a poor prognosis for recurrence
					Tongue-34%				SOHND I-III	Neck recurrence- 19%	Betelnut	
					FOM- 6%					local/neck recurrence - 9%	Tobacco	
					Lip- 1%					local/distant metastasis 3%		
					Buccal- 37%					neck/distant metastasis-14%		
					Gum- 15%					locoregional/distant - 7%		
					RMT- 6%							
Jayasuriya [21]	2020	Sri Lanka	187	72%	OSCC	cN+	NA	I- 58.3%	Neck dissection	NA	NA	Routine MRND not recommended in cN+
					Anterior 2/3^rd^ of tongue- (4/68)			II- 56%				Level V dissection recommended when nodal stages >N2b & metastasis to level III and IV
					Buccal mucosa- (4/68)			III- 40%				
								IV- 27.3%				
								V- 6.4%				
Haranadh [22]	2018	India	199	45%	Buccal mucosa- 171	TNM	Level IIB involvement when IIA involved by 2 or more LN - 40%;	pN0- 125	MRND- 178	NA	NA	Level V not recommended when the primary site is buccal
					Tongue- 15		Level V involvement when level III involved by 2 or more LN 100%	pN1-74	SND I-III- 11			Recommended when level III involved nodes >2, frozen section can help in the decision
					RMT- 6				SND I-IV- 10			
					Lower alveolus- 4			IA-4%				
					Lip- 2		occult metastasis 17%	IB-30%				
					FOM- 1			IIA-14%				
								IIB-3%				
								III- 5%				
								IV-1%				
								V-3%				
Chheda [23]	2014	India	210	74.20%	Tongue-71.4%	TNM	LN metastasis 42 Pts (20%)	IA- 28 Pts	Modified neck dissection- 120 Pts	NA	NA	Routine level IIB not recommended
								IB- 24 Pts				
					Buccal mucosa- 14.2%	cN0		IIA- 16	Extended SOHND- 40 Pts			To be decided on frozen section examination.
					Lower alveolus- 12.3%			IIB- 2 (0.95%)	SOHND- 50 Pts			
					RMT - 1.9%			III- 2				
								IV/V- 0				
Kakei [24]	2020	Japan	100	58%	Tongue-45 Pts	cTN1M0	LN metastasis 66%	pN1:	SOHND	NA	NA	Level V to be excluded, level IV to be considered with Ca tongue and clinical LN metastasis at level II or III
					Lower gingiva-24 Pts	IA-2 Pts		IA-2 Pts				
					Buccal mucosa- 15 Pts	IB-61 Pts		IB-20 Pts				
					Oral floor-8 Pts	II-37 Pts		II-14 Pts				
					Upper gingiva-8 Pts	III-0		III-1Pts				
						IV-0		IV/V-0				
						V-0		pN2b:				
								IA-1 Pts				
								IIB-8 Pts				
								II-10 Pts				
								III-8 Pts				
								IV-2 Pts				
								V-0				
Marchiano [25]	2016	USA	8281	62.30%	OSCC	TNM	N+ve (24.1%)	in T1 : level IV (3.1%) level V (1.1%)	Neck dissection	5 year DSS: with Level I, II, or III involvement - 42%	NA	Level I-III should be routinely dissected in OSCC
					buccal (6.2%)			in T2 : level IV (6.5%) level V (3.1%)				Level IV/V involvment has worse prognosis
					FOM (16.4%)			in T3 : level IV (9.5%) level V (3.7%)				
					gum (9.6%)		distant metastasis (1.6%)			Level IV involvment DSS- 30.6%		
					Hard palate- (2.3%)							
					lip (18%)			in T4 : level IV (11.2%) level V (4.9%)				
					RMT (5.4%)					DSS if level V- 26.4%		
					tongue (42.1%)							
Givi [26]	2012	Canada	108	64%	Mucosal SCC of head and neck	TNM	N+ve - 108 (all Pts)	I-III: (11.1%)	SND	recurrence- 5.5%	NA	SND effective in selected patient groups ( with low-volume disease on preoperative imaging and no ECE)
					Oral cavity- 71.3%			I-IV: (79.6%)		death- 21.3%		
					Oropharynx - 22.2%			II-IV: (4.6%)		DSS- 76.9%		
					larynx - 4.6%			II-V: (4.6%)				
													Pandey [27]	2018	India	32 cN-ve Pts	87.50%	OSCC	TNM 1-4	3 Pts has pN+ level Ib	I-III: 30	IIB preserving super-selective neck dissection (SSND), SOHND	Recurrence- 3 Pts	NA	SSND is safe oncologically in patients with cN-ve
Buccal mucosa- 18	I-IV: 2	DFS- 83% in (SSND)																							
Lower alveolus- 6		DFS - 91% in (SOHND)																							
Tongue-8																									
Agarwal [28]	2018	India	231	82.75%	OSCC	N0	LN mets 30.73%	IIA- 11.68%	SND	local recurrence 2.59%	NA	SND I-III adequate, level IIB & IV dissection not required for N0 patients
					buccal - 50.2%			IIB- 0.86%,		nodal recurrence 9.52%		
					Tongue- 36.3%			IV- 0				
Mishra [29]	2010	India	81	NA	OSCC	T1-2N0M0;	26% (occult)	N0 Cases: Levels I, II, III (26%)	SOHND, Extended SOHND, MRND-I	local recurrence 2 Pts	NA	SOHND recommended for N0 cases, and MRND-I for N+ cases
					Tongue - 34 Pts	T1-3N1M0		Level IV/V- No metastasis				
					buccal -19 Pts			N+ Cases: Level IV-9%		neck recurrence- 0		
								Level V- 0				
					others-28 Pts			Skip metastasis-0				
Shimura [30]	2019	Japan	131	59%	OSCC	TNM 1-4	LN mets 52%	ipsilateral I-VI	SND, MRND/ RND	Primary Recurrence- 28%	NA	In neck nodes positive cases, for up to 2 LNs, SND recommended
					Tongue- 41%			contralateral I-IV		OS (cN0)- 80%		
					lower gum - 22%					DSS (cN0)- 88%		
Parikh [31]	2013	India	210	155	Buccal mucosa- 43%	TNM	cN0 - 23% (occult metastasis)	Level V- 4.3%	SND	NA	NA	SND recommended for Cn0 and cN1 occurring with level Ib
					Tongue/FOM- 31%		cN+ve - 77%	Ib- 99/112				
					Alveolar- 12%			II/III- 13/112				
					Gingivobuccal- 10%			Skip metastasis- 0				
					Lip- 4%							
Jerjes [32]	2010	UK	115	56.50%	OSCC:	T1-2N1-2M0	pN1 - 12 Pts	NA	Primary resection + neck dissection	Recurrence- 37.4%	NA	Not described
					FOM- 20.9%							
					Tongue- 46.9%							
					Buccal mucosa- 2.6%		PN2 - 22 Pts			5-year survival- 72.2%		
					Alveolus Retromolar area- 2.6%							
					Lower lip- 4.5%							
Cariati [33]	2018	Spain	53	29	Buccal mucosal squamous cell ca	T1-T4	LN metastasis 17 Pts (32%)	IB-59.3%	NA	Recurrence- 67.9%	Tumor stage and thickness, N stage	Recommend SOHND for early T buccal ca
						N0, N1, N2		IIA- 30.5%		5-year survival- 69.8%		
								IIB- 0				
								III- 10.1%				
								IV- 0				
								V- 0				
Patel [34]	2019	India	30	24 Pts	OSCC	T1-T4	LN metastasis - 36.7%	level I- 50%	MRND, RND, SOHND	NA	Tobacco chewing	SOHND & MRND appropriate for N0 and N+ oral cancer cases
					Buccal- 36.7%			II- 28.57%				
					Tongue- 30%			III- 11.9%			alcohol	
					Alveolus- 20%			IV -7.14%			betelnut	
					Bucco-alveolar- 10%			V- 2.38%			smoking	
					Lower lip- 3.3%			Skip III- 6.7%				
								Skip IV- 0				
Lodder [35]	2008	Netherlands	291	NA	oral and oropharyngeal carcinoma	T1-T4 / N0, N1	Oral cavity (201 Pts )	Level III- 4%	MRND I-V - 60%	NA	NA	SOHND I-III recommended for routine, Inclusion of lower levels not recommended
													Skip metastasis (III/IV)- 6%	Level IV (in N0/N1)- 2%
							LN metastasis- 48%	Level IV (in N2)- 26%				
								level V (in N0/N1) - 2%					SND I-IV - 40%	In N2 patients level, IV should be included
								level V (in N2) - 5%						
								level V ( in N3) - 20%						
							Lim [36]	2006	Korea			93	80 Pts	oral/ oropharyngeal SCC	N+ve	LN metastasis -91%	level I- 17%	Comprehensive Neck dissection	NA	NA	Multiple neck nodes significantly associated with metastasis level V (P=0.023)
level II- 70%																					
level III- 41%																					
occult metastasis level V - 4%	level IV- 31%																				
	level V ipsilateral -5%	Level V to be preserved below N2a level in N+ OOSCC																		
	level V contralateral - 0%																			
Kowalski [37]	2002	Brazil	164	86.60%	oral cavity ca	T1-T4 /cN1,cN2a	LN mets 57.9%	level I - 8.5%	RND	regional recurrence- 8.5%	NA	SOHND appropriate for N1, N2a
					Tongue- 43.9%			level II 35.4%				
					Floor of the mouth- 23.8%			level III - 2.4%				
					retromolar - 16.5%			Level IV- 0.6%				
					buccoalveolar sulci- 3.7%			level V- 0%				
					lower gum - 12.2%			multi-levels- 11.6%				
Feng [38]	2013	China	637	55.40%	OSCC	N0, N+ve	occult metastasis 28.4%	I- 55.1%	SOHND, RND/ MRND	neck recurrence- 9.2%	NA	SOHND appropriate for OSCC N0, ESOND also an alternative in N+
								II- 38.2%				
								III- 6.7%				
								Skip metastasis Level IV/V- 0%				
Sivanandan [39]	2004	USA	100	74 Pts	oropharynx & oral cavity- 80%	N0-N3	LN 25%	I-IV	RND, MRND	N2-N3 neck disease- 59 Pts	NA	No recommendation
										Neck Recurrence- 7% ( after radiotherapy 4% )		
Crean [40]	2003	UK	49	24 Pts	oral cavity	N0	LN 26.5%	Level IV occult metastasis- 10%	ESOHND	neck recurrence- 8.2%	NA	ESOHND recommended for N0 necks
					FOM 16 Pts							
					Tongue 14 Pts							
Khafif [41]	2001	USA	51	NA	Oral Tongue	T1-T3/ N0	occult metastasis 26%	Level IV mets 4%	Neck dissection I-III, and IV	16% neck recurrence	NA	SOHND is enough for tongue T1-T3 / N0
Balasubramanian [42]	2012	India	52	43 Pts	Oral Tongue	T1-T4, N0-N2	LN mets 39.5% (17 Pts)	Level III skip mets- 3.8%	Neck dissection	Recurrence- 3 Pts (1 in neck)	NA	SND is enough for N0 early stages T1/T2
								Level IV skip mets- 1.9%				
Köhler [43]	2018	Brazil	163	89.57%	tonsillar SCC	T1-T4	6% (levels IV-V)	Combinations present for levels	SND	neck recurrence -12 Pts	Tobacco	In cN0 patients, removal at levels II and III is mandatory but levels I, IV, and V may be spared
									MRND	Deaths-61 Pts	Alcohol	
Deo [ 44]	2019	India	945	77.57%	Buccal mucosa-28.78%	T1-T4	LN mets- 39.7%	Skip metastasisLevel III-5%	Modified neck dissection	NA	Tobacco chewing	Inconclusive on the inclusion of lower levels
					Tongue- 21.16%	cN0		skip metastasis Level IV-2%				
					Alveolo-buccal-18.73%			skip metastasis Level V-0.5%				
					Alveolus- 11.01%				SOHND		Smoking	
					Central arch and FOM- 9.52%	cN+						
					RMT- 5.71%							
					Lip- 5.08%							
de Vicente [45]	2015	Spain	56	75%	Tongue- 35.7%	TNM	LN mets 51.8%	IIb	SND (I-III)	Recurrence-7.1%	Tobacco, alcohol	Recommend dissection of level IIB only if multilevel involvement or level IIA involved
					Floor of the mouth-23.2%				ESND (I-IV)	Survival (without recurrence)- 80.4%		
					Gum- 23.2%				MRND (I-V)			
					Palate- 3.6%				RND			
					Buccal- 3.6%							
					Retromolar- 10.7%							
Rani [46]	2015	India	10	60%	Lower alveolar ridge- 50%	TNM	LN mets 50%	I & II	SND (I-III)-6 Pts	Survival-70%	NA	No recommendation
					Upper alveolar ridge-10%							
					Buccal mucosa-10%				MRND-4 Pts	regional recurrence 20%		
					Tongue-20%							
					RMT-1%							
Chatterjee [47]	2019	India	126	104 Pts	anterior two-thirds of tongue- 52.2%	TNM	LN mets 38.1%	N0- 78 Pts	NA	Recurrence-2 (2/48)	NA	Tumor budding and pattern of invasion are associated with a higher risk of cervical LN metastasis
					buccal mucosa- 36.2%			N1-18 Pts		Died- 8 (8/48)		
					others- 11.6%			N2b- 28 Pts				
								N3b- 2 Pts				
Vishak [48]	2014	India	57	75.40%	Oral Tongue	TNM (T1)	LN mets 36.8%	I- 10.5%	MRND	NA	higher grade, tumor size >1 cm	Oral tongue ca with Tumor thickness >3mm associated with a higher risk of LN metastasis
								II- 10%				
								Skip metastasis to III-IV 8.5%				
								Skip metastasis to IV 1.75%				

The prevalence of metastasis ranged from 1.8% to 66.0% [24]. Among 23 studies reporting metastasis level up to level V, 13 studies [19-22,24,29,34,35,37,40-43] reported level IV involvement, and eight reported level V involvement [19-22,31,34,36,43]. The rate of involvement of level IV among the patients with cN0 was up to 10.4% [19], with four studies [23,28,29,33] reporting no involvement.

Six articles [19,29,31,34,38,48] illustrated the characteristics of cervical skip metastasis patients, which gave details of sites, T stages, isolated IIb metastases [45], and associated metastatic lymph nodes. The incidence for skip metastasis to level IV or V was low, reaching up to 8.5% [29,31,34,48]. However, not all the information was complete for each study. The most common primary site for level IIb metastases was the tongue [22-24,45,47], reported between 2% and 28% [23,47]. The rate of skip metastasis among cN0 was also low, reaching 1.8% [19,29,31].

Studies Recommending Dissection of Lower Levels

Five studies [17,21,24,45,48] recommended dissection of lower neck levels. Three of these studies [21,24,48] reported metastasis to level IV, while one [17] reported metastasis to level V. None of them were on patients with cN0, two [21,24] had data on N+, while three [17,45,48] had mixed data. One study reported metastasis to level IIb in tongue carcinoma [45].

Studies Not Recommending Dissection of Lower Levels

Thirteen studies [21,22,24,28-31,35-37] did not recommend dissection of lower neck levels because of the low prevalence of metastasis to these levels. Only six of these studies [28-31,35,37] reported metastasis to level IV, while five studies [21,22,24,35,36] reported metastasis to level V. Four of them were on patients with cN0 [23,28,29,31], while six [21,24,29-31,36] presented data on N+ patients. Three studies [22,35,37] reported mixed nodal status, and one study [23] was on level IIb involvement for oral tongue carcinoma.

Studies With Inconclusive Results on Dissection of Lower Levels

Few studies [18,19,34,39,41,47] were inconclusive in recommending whether lower-level dissections should be undertaken or not, with routine neck dissections. These studies reported no metastasis at level IV or V and concluded that SND I-III was sufficient in most cases. However, these studies also went on to recommend dissection of levels IV and V based on the surgeons’ clinical decisions during surgery. Of these, one [19] reported data on cN0 neck, one [18] on N+ neck, and four [34,39,41,47] had mixed nodal status. In addition, twelve studies [20,25-27,32,33,38,40,42-44,46] did not make any clear recommendation on inclusion or non-inclusion of lower levels for neck dissections for lack of such data. A study by Jayasuriya et al. [21] presented ambiguous results wherein the authors did not recommend routine neck dissection for level V; however, they went on to recommend level V dissection when nodal stages >N2b and metastasis to level II and IV were observed in a case.

Discussion

This review revealed that the available literature favored either selective neck dissection, including only the upper levels (I-III), or was inconclusive. Most studies support the view that primary neck dissections should be limited to upper levels only, owing to the low rates of lower level (level IV and beyond) metastasis and the difficulty as well as the damage incurred (thereby introducing complications) due to the inclusion of those levels. Through independent studies, most authors have supported that high efficacy and minor morbidity for selecting pN+ OSCC patients may be achievable using SND (I-III) [38,49,50]. In a meta-analysis that compared SND with MRND/RND in OSCC patients with cN+ disease, authors [51] suggested that cN+ OSCC patients treated with SND (I, I-III, or I-IV) or those treated with MRND/RND had comparable clinical outcomes measured by no significant difference for regional recurrence, overall survival (OS), or disease-specific survival (DSS) between any of the dissection treatment types. The meta-analysis was, however, limited by the inclusion of studies where the extent and selection of the SND levels differed between studies other than levels I-II. The result of this meta-analysis supports our claim that even with variable surgical methods, it is not advisable to routinely include lower-level dissections. Contrary to the findings of the present study, independent studies, such as one by Shah et al. [52], have reported that 15%-16% of tongue/oral cancer with clinically detected lymph node(s) (cLN(s)) had pathological lymph node(s) (pLN(s)) to level IV, thereby recommending extended SOHND, which includes dissecting level IV.

Skip metastasis, described by Byers et al. [14], refers to the condition in which OSCC bypasses levels I, II, or both and goes directly to levels III or IV. The rate of skip metastasis in the original study was reported as 15.8%, thereby recommending routine dissection at neck level IV. Later analysis, however, revealed that among cN0 patients, only 5.5% had skip metastasis to level IV, making the recommendations controversial. Later, Crean et al. [40] similarly demonstrated that 10% of patients had involvement of neck level IV despite having been preoperatively diagnosed with a cN0 neck, with only 2% having a true skip metastasis to level IV. In a recent meta-analysis, the authors found the rate of skip metastasis to be low (overall involvement rate of 2.53% and skip metastasis rate of 0.50%), even with advanced tumor stages, wherein the final recommendation was not to include dissection of lower levels routinely [53]. A meta-analysis was conducted in 2020 to investigate the prevalence of level IV involvement and skip metastases in patients with clinically negative neck (cN0) oral tongue squamous cell carcinoma. It also recommended elective neck dissection that includes levels I to III because of the low rates of level IV involvement and skip metastasis [54]. Our review also supports the view for non-inclusion of lower levels in ND for suspicion of skip metastasis.

Some arguments may be made in terms of benefits archived in ipsilateral, contralateral, or bilateral node infiltration. Although we did not study the laterality of recurrence, the available literature [30] suggested that SND (I-III) could achieve good regional control and had a favorable prognosis for cN+ OSCC. In a study with ipsilateral neck recurrence rates ranging from 11%-14%, similar conclusions were drawn for the pN+ cohort [30].

Some studies reported data on oral tongue SCC, which is the most common primary site for OSCC, with most studies suggesting metastasis to level IIb [55,56], leading scholars to recommend level IIB dissection routinely in tongue SCC. Few studies [57,58] found no statistical significance between site and metastasis, which makes a contrary view due to the difficulty of approach, questionable benefits, and avoidance of postoperative shoulder disability [8]. Even with regards to level IV metastasis, most studies present a reserved view to include lower-level dissection as an exception for tongue carcinoma [14]. Our study found that all included literature for oral tongue carcinoma recommended lower-level dissection, probably owing to the tendency of tongue cancer toward early metastasis, the possible reason being that the tongue possesses an extensive lymphatic network.

Strengths and limitations

The present review included studies that reported varied study groups and regions, thereby introducing heterogeneity. The heterogeneity of study groups is considered an important confounder. In our case, it resulted in the lack of appropriate data stratification by T stage, subsites, and involvement of other neck levels that we could not address. The retrospective nature of the included studies also introduced bias, which could not be addressed. However, we exercised caution in including studies with primary neck dissection data only. We excluded all studies with patients with revision NDs and omitted all groups lacking this information to eliminate bias from combining the results of the primary neck surgery with those of revision surgeries for neck recurrences, which may falsely inflate the rate of level IV or lower-level involvement. While most studies presented mixed data for cN0 and cN+ necks, we segregated data wherever possible to report the differences according to nodal status. Lastly, the decision for SND or MRND techniques is widely debated due to the lack of universally accepted guidelines for the anatomic limits for the variety of SND procedures available. The exact anatomic boundaries for an SND are also thought to vary among institutions and even among surgeons within an institution [59]. The analysis of these differences could not be accounted for in the present review.

## Conclusions

OSCC is constituted by a broad range of tumors with diverse etiologies. It can metastasize to cervical lymph nodes via lymphatic vessels. SND is considered a standard of care for most subsites, even in early-stage disease. Based on the evidence reviewed in the present study, the frequency of lower-level metastasis (level IV or V), as well as skip metastasis in OSCC, was low. Hence, routine dissection of these levels in cN0 and cN+ necks may be avoided except for tongue cancer. Since dissection of level IV/V is a burden with extra time and might expose patients to more complications, dissection might be selected for specific subsites and extension. It is recommended to dissect level IIb and lower levels for tongue cancers without considering the stage of primary lesions or lymph node status. Most studies recommended sparing lower-level neck dissections, while some were inconclusive.
